# Inverse Regulation of TLR4 and PD‐L1 Shapes the Inflammatory Tumor Microenvironment in Oral Squamous Cell Carcinomas

**DOI:** 10.1111/jop.70012

**Published:** 2025-08-05

**Authors:** Camila Alves Ferri, Giuseppe Pannone, Maria Carmela Pedicillo, Giorgio Mori, Ilenia Sara De Stefano, Francesco Angelillis, Raffaele Barile, Roberta Seccia, Silvana Papagerakis, Angela Santoro, Gian Franco Zannoni, Gabriella Aquino, Margherita Cerrone, Monica Cantile, Vito Rodolico, Giuseppina Campisi, Renato Franco, Francesco Miele, Rosanna Zamparese, Francesco Longo, Lorenzo Lo Muzio, Fernanda Visioli

**Affiliations:** ^1^ Oral Pathology and Oral Medicine Department, School of Dentistry Universidade Federal Do Rio Grande Do Sul Porto Alegre Brazil; ^2^ Department of Clinical and Experimental Medicine Pathological Anatomy Unit, University of Foggia Foggia Italy; ^3^ Department of ENT‐Head and Neck Surgery Faculty of Medicine, Université Laval Quebec Canada; ^4^ Department of Sciences of the Women and Child Health Operative Unit of Gynecological and Breast Pathology, Fondazione Policlinico Agostino Gemelli – UCSC Rome Italy; ^5^ Department of Pulmonary Oncology AORN Dei Colli Monaldi Naples Italy; ^6^ Pathology Unit, Istituto Nazionale Tumori – INT‐ IRCCS – Fondazione G. Pascale Napoli Italy; ^7^ Department of Sciences for the Promotion of the Maternal and Childhood Health “G. D'alessandro”, Section of Anatomic Pathology University of Palermo, A.O.U. Policlinico “P. Giaccone” Palermo Italy; ^8^ Departament of Surgical, Oncological and Oral Sciences University of Palermo Palermo Italy; ^9^ Pathology Unit, Department of Mental and Physical Health and Preventive Medicine University of Campania Luigi Vanvitelli Naples Italy; ^10^ Department of Surgery University of Campania Luigi Vanvitelli Naples Italy; ^11^ Legal Medicine Unit, Ascoli Piceno Hospital C‐G Ascoli Piceno Italy; ^12^ Department of Clinical and Experimental Medicine Oral Pathology Unit, University of Foggia Foggia Italy

**Keywords:** CD8, mortality, oral squamous cell carcinoma, PD‐L1, TLR4, tumor infiltrating lymphocytes (TIL), tumor microenvironment

## Abstract

**Background:**

The interactions between malignant cells and immune cells within the tumor microenvironment (TME) significantly influence cancer development and progression. This study aimed to analyze and correlate the expression of TLR4 and PD‐L1 with the immune response, clinical characteristics, and prognosis of oral squamous cell carcinomas (OSCC).

**Methods:**

Our retrospective multicentric study consisted of the assessment of 166 OSCC specimens for TLR4, PD‐L1, CD8, and Ki‐67 expression in a TMA‐based immunohistochemistry analysis.

**Results:**

Our findings indicated an inverse correlation between the expression of PD‐L1 and TLR4 (r = −0.348, *p* = 0.014, and r = −0.269, *p* = 0.049, superficial tumor site and in overall analysis, respectively). On the other hand, PD‐L1 expression in the deep and superficial invasive front positively correlated with CD8+ T tumor infiltrating lymphocytes (TIL) in a statistically significant manner. A logistic regression analysis was performed to assess the impact of each variable on the clinical outcome with at least 5‐year follow‐up after the initial OSCC diagnosis. The multivariate model revealed that advanced T stage (T3‐T4), presence of lymph node metastasis (N+), as well as performing chemotherapy were statistically significantly associated with OSCC mortality.

**Conclusion:**

These findings taken together suggest that there is a differential regulation of the immune response coordinated by activation of PD‐L1 or TLR4 affecting T cell response.

## 1 Introduction

Oral squamous cell carcinoma (OSCC) affects the lip mucosa, gingiva, buccal mucosa, tongue, and other oral cavity structures [1]. Worldwide, it ranks 16th among global cancers, with 389 485 cases and 188 230 deaths reported in 2022 [2]. Key risk factors include alcohol and tobacco consumption, which can synergistically increase cancer risk through field cancerization. Additionally, human papillomaviruses (HPV) are involved in the etiopathogenesis of a subgroup of OSCC cases. Other contributing factors include age, gender, socioeconomic status, genetic predispositions, hormonal influences, and other viral infections [3].

The tumor microenvironment (TME) of solid tumors, including OSCC, significantly influences cancer development and progression through interactions among malignant, inflammatory, and stromal cells. Inflammatory responses are critical across tumor stages, including initiation, promotion, malignant transformation, invasion, and metastasis [4]. Studies show that recruitment, activation, and reprogramming of immune and stromal cells, along with their interactions with cancer cells in the TME, directly impact clinical outcomes and responses to conventional and targeted therapies in OSCC patients [4, 5].

Recent evidence highlights the pivotal role of CD8+ T infiltrating lymphocytes in immune anticancer defense mechanisms. CD8+ T lymphocytes detect and eliminate aberrant cells expressing tumor‐specific antigens, playing a crucial role in cancer suppression. They also maintain immunological memory through a persistent post‐activation state, preventing recurrences [6]. As key immune system components, CD8+ cells are vital for cancer surveillance and eradication, making them targets for therapeutic interventions that have spurred innovative immunotherapy strategies [6]. The PD‐1/PD‐L1 axis, a critical pathway in cancer immunity, suppresses lymphocyte proliferation and activation when imbalanced, weakening immune capacity [7]. Within the TME, PD‐1/PD‐L1 interactions facilitate immune evasion, impair T cell function, and exhaust PD‐1‐expressing CD8+ T cells, reducing their ability to eradicate cancer [8]. Immune checkpoint inhibitors have shown promise in treating cancers, highlighting PD‐L's role as a therapeutic target in oncology [5].

Toll‐Like Receptors (TLRs), key components of innate immunity, influence cell–cell communication within the TME through their expression on tumor and immune‐associated cells. These transmembrane proteins, found on immune sentinel cells like macrophages and dendritic cells, are expressed on the cell surface or intracellularly [9]. TLR expression and its anti‐tumor effects in immune checkpoint blockade have been extensively studied in cancer [10]. Among the 10 identified TLRs, TLR4 is linked to tumor progression and antitumor immunity [11]. TLR4 activation mediates tumor resistance to cytotoxic T lymphocytes, promoting tumor growth and immunosuppression in vivo and within the TME [11, 12]. Its tumor‐promoting roles are well‐established in colon, liver, pancreas, and skin cancers [10], but few studies have explored TLR4 involvement in OSCC [13, 14]. Understanding TLR and PD‐L1 interactions in the TME is crucial for developing therapies to restore antitumor immunity. While TLR4‐PD‐L1 correlations have been studied in lymphomas [15] and lung cancers [16], their role in OSCC remains unclear. This multicentric retrospective study analyzes TLR4 and PD‐L1 expression, immune responses, clinicopathological parameters, and prognoses in 166 OSCC cases.

## 2 Materials and Methods

### 2.1 Patient Population

This is a retrospective multicentric population‐based study involving different Italian oncology centers. Permission from the Institutional Review Board (IRB) for Human studies of the respective ethics committees was granted from all participating centers to retrospectively analyze their OSCC specimens' collections. This study was performed in accordance with good clinical practice guidelines and the Declaration of Helsinki. All the cases included in this study were diagnosed and treated according to national standardized guidelines.

Cases were retrieved from the Pathology Units of the University of Rome (Università Cattolica del Sacro Cuore—Fondazione Policlinico Universitario A. Gemelli Scientific Institute for Research, Hospitalization and Healthcare—IRCCS), University of Palermo (Azienda Ospedaliera Universitaria A.O.U. Policlinico “P. Giaccone”), and University of Naples INT‐IRCCS Fondazione “G Pascale” and University of Campania Luigi Vanvitelli.

This study included 166 primary OSCC surgical specimens that were routinely formalin‐fixed and paraffin‐embedded (FFPE) between 1996 to 2006. Two anatomo‐pathologists (G.P. and I.S.DeS.) independently examined and staged each OSCC specimen according to the TNM classification AJCC 8th edition 2017 [1]. Tissue slides were reviewed to confirm the original diagnosis, and clinicopathological characteristics were collected, including age, gender, tumor location, T‐stage, N‐stage, tumor grading differentiation according with standardized Broder classification. All patients underwent surgery in association or not with other adjuvant therapies (radiotherapy and/or chemotherapy).

The TMA was generated by collection of two 0.6 mm core biopsies from the superficial (s) and the deep (d) invasion front. The invasion front was assessed according to the same AJCC classification [1].

### 2.2 Immunohistochemistry

TMA‐based immunohistochemistry (IHC) was performed on 4 μm thick paraffin sections mounted on poly‐L‐lysine‐coated glass slides, by automated linked streptavidin‐biotin horseradish peroxidase (LSAB‐HRP) technique, performed by Ventana Benchmark XT autostainer, using a specific monoclonal antibody against the following: TLR4 (NOVUS BIOLOGICALS, Centennial, CO, USA, clone 76B357.1, dilution 1:300), PD‐L1 (clone SP142, ROCHE—VENTANA/CELL MARQUE, Rocklin, CA, USA, prediluted), CD8 (clone SP57, ROCHE—VENTANA/CELL MARQUE), Ki‐67 (clone 30–9, ROCHE—VENTANA/CELL MARQUE). Gill's type II hematoxylin was used for nuclear counterstaining. Appropriate corresponding positive and negative controls were run for each tested antibody. Sections were digitally scanned with NanoZoomer S60 C13210 series Hamamatsu Photonics K.K., Japan and using open NDP.view2 Image viewing software, U12388‐01.

TRL4 was evaluated on the basis of percentage (0–100) of any membranous‐cytoplasmic positivity both in superficial (s) and deep (d) tumor areas, and also as an average of the whole sampling. PD‐L1 was assessed using tumor cell score (TC), which represents the proportion of PD‐L1‐positive tumor cells as a percentage of the total tumor cell count [17], both in superficial (s) and deep (d) tumor areas, as well as the average in the whole tissue. The assessment of immune infiltration levels within the tumor microenvironment was performed by quantifying the density of CD8+ cells [cells/mm2]. As performed for the other markers, CD8 was assessed on (s) and (d) areas and assessed the average on the tissue. The Ki‐67 protein expression was evaluated as a percentage (0–100) of any nuclear staining in neoplastic cells.

### 2.3 Statistical Analysis

The normality of the data was assessed using the Kolmogorov–Smirnov and Shapiro–Wilk tests, as well as by examining data histograms. A non‐Gaussian distribution was observed; therefore, non‐parametric tests were employed in the analyses. The Kruskal‐Wallis and Mann–Whitney U tests were used to compare the distribution of immunohistochemical markers across different variables. Spearman's rank correlation and the chi‐square test were performed to evaluate associations between the data and various clinicopathological and histological variables. All statistical analyses were conducted using SAS software, version 9.2 (SAS Institute Inc.). A two‐tailed *p* ≤ 0.05 was considered statistically significant. Logistic regression analysis was performed to assess the impact of clinicopathological factors and marker expression on clinical outcomes and mortality status, using the IBM Statistical Package for the Social Sciences (SPSS, version 22; Armonk, NY: IBM Corp).

## 3 Results

### 3.1 Cohort Characteristics

The clinicopathologic characteristics of this cohort of 166 primary OSCC cases are summarized in Table 1. The majority of cases were prevalent in male patients (64.45%), with a male: female ratio of 2:1. The average age at the time of diagnosis was 67 ± 12 years, ranging from 31 to 79 years. The most frequent anatomical site of the primary tumors was the tongue (48.8%), followed by the floor of the mouth (11.4%). All patients underwent surgical treatment. Regarding the adjuvant therapies, some patients received radiotherapy (34.9%), some chemotherapy (1.2%), and the remaining received both (18%).

**TABLE 1:** Clinicopathological characteristics of the OSCC cohort (*n* = 166).

Characteristics		*n* (%)
Age range years old (Mean ± SD)	31–79 (67 ± 12)	—
Gender	Female	47 (28.3%)
	Male	107 (64.5%)
	Missing information	12 (7.2%)
Primary tumor anatomic subsite	Tongue	81 (48.8%)
	Floor of mouth	19 (11.4%)
	Multiple locations	14 (8.4%)
	Gum	12 (7.2%)
	Trigonus	10 (6.0%)
	Multiple locations in tongue	9 (5.4%)
	Lip	4 (2.4%)
	Buccal mucosa	3 (2.0%)
	Missing information	14 (8.4%)
Tumor dimension range cm (Mean ± SD)	0.3–7 cm (2.8 ± 1.34)	—
Deep of invasion (DOI) range mm (Mean ± SD)	1–24 mm (11 ± 4.98 mm)	—
Grade	G1	25 (15.06%)
	G2	78 (46.98%)
	G3	33 (19.87%)
	Missing information	30 (18%)
T stage	T1	28 (16.7%)
	T2	71 (42.8%)
	T3	27 (16.3%)
	T4	26 (15.7%)
	T4a	1 (0.6%)
	Missing information	13 (7.8%)
N stage	N0	76 (45.8%)
	N1	31 (18.67%)
	N1a	1 (0.60%)
	N2	10 (6.02%)
	N2b	22 (13.25%)
	N2c	14 (8.43%)
	Missing information	12 (7.22%)
M stage	M1	1 (1.0%)
	M0	152 (92.0%)
	Missing information	12 (7.0%)
HPV status (p16)	Negative	59 (55.1%)
	Positive	48 (44.9%)
	Missing information	47 (28.3%)
Rxt and/or Chm	Rxt	58 (34.9%)
	Chm	2 (1.2%)
	Rxt + Chm	30 (18%)
	No adjuvant therapy	31 (18.67%)
	Missing information	45 (27.1%)
OSCC mortality	Alive	51 (30.7%)
	Death	53 (31.92%)
	Missing information	62 (37.34%)

Abbreviations: Chm = chemotherapy, Rxt = radiotherapy.

The correlation between clinicopathological parameters of the cohort was evaluated by Spearman's Rank Coefficient test (Table 2). From this analysis emerged a direct correlation with depth of invasion (DOI) and tumor dimension (*r* = 0.374, *p* = 0.001), N (*r* = 0.222, *p* = 0.007), T (*r* = 0.602, *p* < 0.001), and tumor stage prognostic groups (*r* = 0.361, *p* < 0.001). Tumor dimension was also associated with T (*r* = 0.457 *p* < 0.001) and tumor stage (*r* = 0.296, *p* = 0.011). N was directly associated with T (*r* = 0.278, *p* < 0.001) and tumor stage (*r* = 0.812, *p* < 0.001).

**TABLE 2:** Correlations between clinicopathological parameters of the OSCC cohort evaluated by Spearman's Rank Coefficient test.

		G	DOI	Dimension	N	T	Stage	Age
G	Correlation coefficient	1	−0.004	0.074	0.129	0.017	0.077	0.134
	*p*		0.965	0.536	0.111	0.833	0.344	0.117
DOI	Correlation coefficient	−0.004	1	0.374**	0.222**	0.602**	0.361**	0.113
	*p*	0.965		**0.001**	**0.007**	**< 0.001**	**< 0.001**	0.197
Dimension	Correlation coefficient	0.074	0.374**	1	0.149	0.457**	0.296*	0.071
	*p*	0.536	**0.001**		0.208	**< 0.001**	**0,011**	0.595
N	Correlation coefficient	0.129	0.222**	0,149	1	0.278**	0.812**	−0,182*
	*p*	0.111	**0.007**	0,208		**< 0.001**	**< 0.001**	**0.032**
T	Correlation coefficient	0.017	0.602**	0.457**	0.278**	1	0.621**	−0.004
	*p*	0.833	**< 0.001**	**< 0.001**	**< 0.001**		**< 0.001**	0.96
Stage	Correlation coefficient	0.077	0.361**	0.296*	0.812**	0.621**	1	−0.111
	*p*	0.344	**< 0.001**	**0.011**	**< 0.001**	**< 0.001**		0.193
Age	Correlation coefficient	0.134	0.113	0.071	−0.182*	−0.004	−0.111	1
	*p*	0.117	0.197	0.595	**0.032**	0.96	0.193	

*Note*: Bold values are statistically significant.

Abbreviations: Age = younger than 65 years old versus older than 65 years old; Dimension = maximum size of primary tumor (< 2 cm vs. > 2 cm); DOI = deep of invasion (< 10 mm vs. > 10 mm); G = grade (G1 vs. G2/G3); *p < 0.05; **p < 0.001; Stage = I‐II versus III‐IV; T = T1‐T2 early‐stage versus T3‐T4 advanced stage.

### 3.2 Expression Analysis of Markers in Tumor Specimens

**FIGURE 1:**
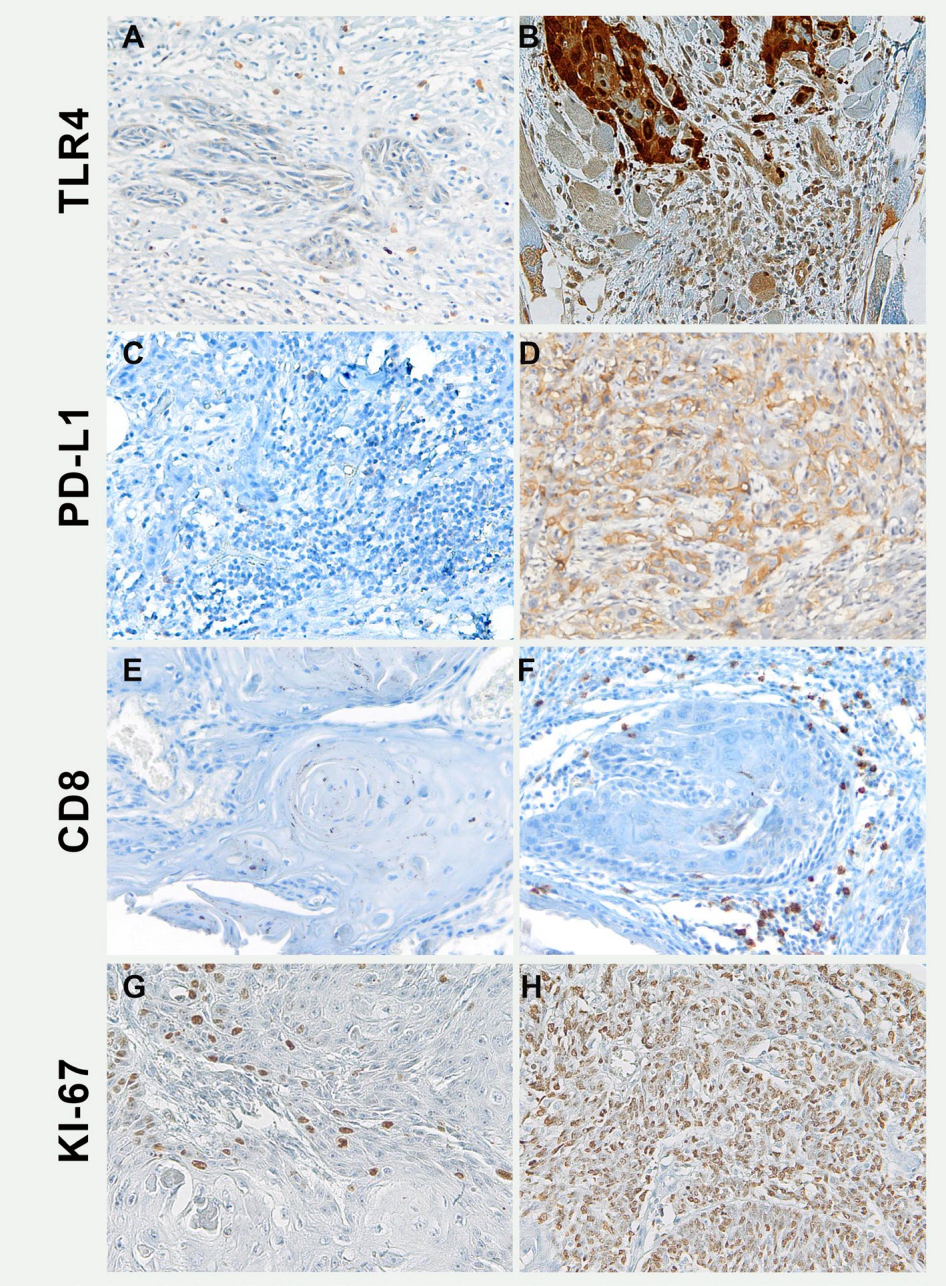
Immunohistochemical biomarkers panel in OSCC: (A) picture shows fainth/negative TLR4 expression in the tumor cells, TLR4 positivity is observed in inflammatory cells surrounding tumor. (B) Image shows strong cytoplasmic expression of TLR4 in tumor cells. (C) picture shows a negative staining for PD‐L1 in tumor cells, and (D) strong immunoreactivity for PD‐L1 in tumor cells of another OSCC sample. (E, F) Immunohistochemical CD8 expression in OSCC in Tumor‐Infiltrating Lymphocytes (TILs) in Grade 1 (E) and in Grade 2 tumors (F). (G) Ki‐67 expression in low‐grade OSCC: Low proliferative activity with minimal Ki‐67 staining. (H) Ki‐67 expression in moderate/high‐grade OSCC: Increased proliferative activity with widespread Ki‐67 staining. Original magnification 20 × NanoZoomer S60 C13210 series.

**FIGURE 2:**
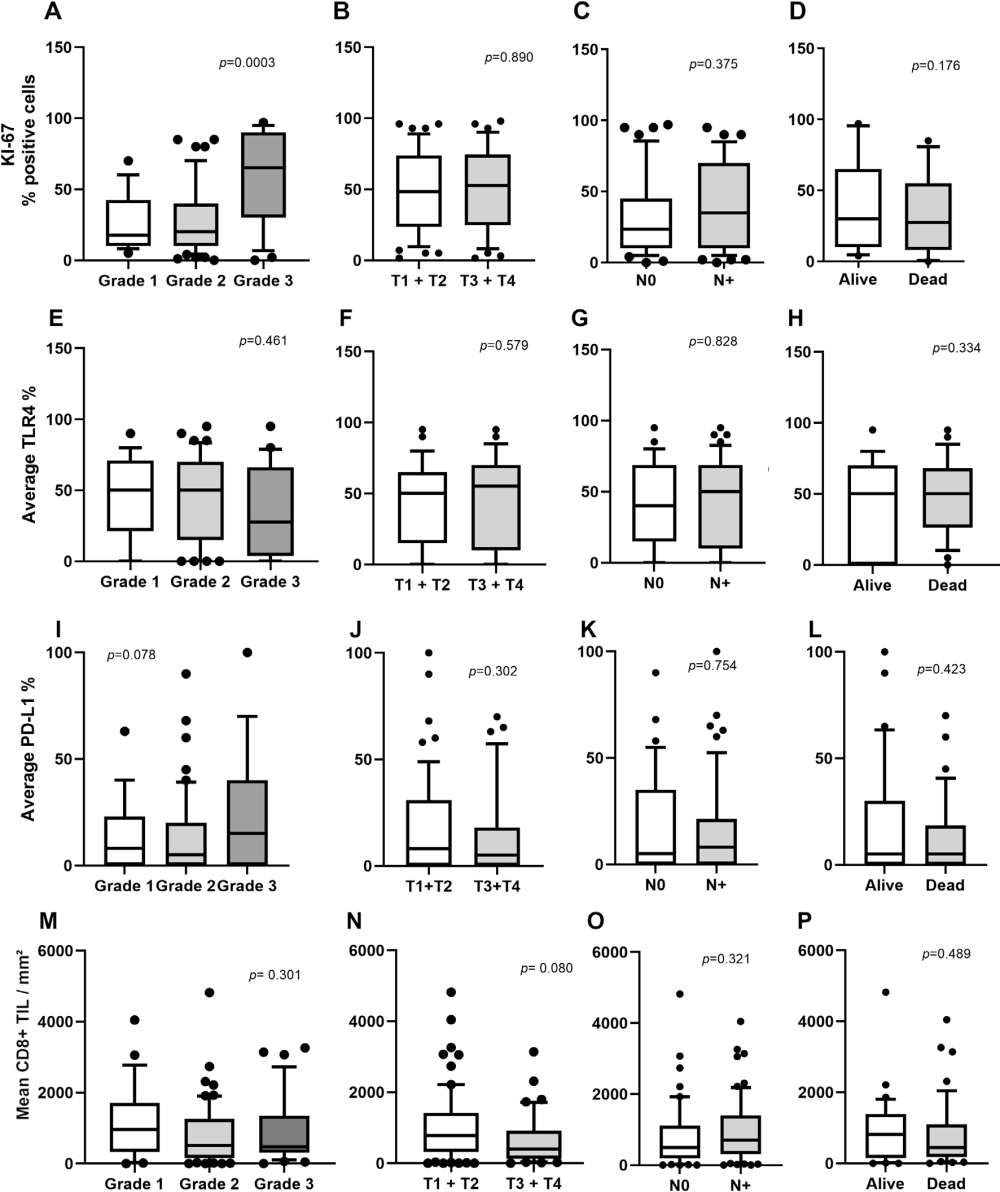
The expression levels of all markers were analyzed in 166 OSCC cases, grouped according to clinicopathological features. (A–D) Percentage of Ki‐67 positive cells according to tumor grade, tumor size (T), nodal status (N), and mortality status, respectively. (E–H) Average number of TLR4‐positive tumor cells according to tumor grade, T size, N status, and mortality status, respectively. (I–L) Average number of PD‐L1‐positive cells according to tumor grade, T size, N status, and mortality status, respectively. (M–P) Quantification of CD8^+^ tumor‐infiltrating lymphocytes (TILs) according to tumor grade, T size, N status, and mortality status, respectively.

Representative images of the marker's staining are shown in Figure 1. The expression levels of all markers were analyzed in 166 OSCC grouped according to clinical‐pathological findings (Figure 2). Specific quantification of each marker in the superficial and deep tumor areas is shown in Figures [Link], [Link]. The analysis demonstrated that Ki‐67 showed an association with tumor grade (*p* = 0.003). Overall expression of PD‐L1 was also increased in G3 tumors, reaching borderline statistical significance (*p* = 0.07), specifically the PD‐L1 (d) in deep tumor invasion (*p* = 0.061). TLR4 expression levels did not show a statistically significant association with other variables. The expression of CD8+ cells was higher in smaller tumors, particularly with the deep front of invasion (*p* = 0.037, Figure S3F) highlighting the importance of immune evasion in tumor progression.

Subsequently, to investigate the interaction between markers and how they may contribute to tumorigenesis, we performed Spearman's Rank Coefficient test (Table 3). Our analysis revealed a positive statistically significant correlation between Ki‐67 status and tumor grade (*r* = 0.366, *p* < 0.001). Interestingly, a general positive correlation was observed between the levels of PD‐L1 expression and CD8+ cells, both in (s) and (d) invasion tumor sites. This generalized trend supports the hypothesis that PD‐L1 may influence the modulation of the inflammatory microenvironment. Moreover, an inverse correlation was observed between TLR4 expression in deep tumor invasion front and PD‐L1 average expression (Figure 1), as well as with PD‐L1 in the superficial tumor front (*r* = −0.348, *p* = 0.014 and *r* = −0.269, *p* = 0.049, respectively).

**TABLE 3:** Correlations among immunohistochemical markers of the OSCC cohort evaluated by Spearman's Rank Coefficient test.

		Grade	DOI	Dimension	N	T	Stage	Age	PDL‐1 (d)	PDL‐1 (s)	Average PDL1	Ki67%	TLR4 (d)	TLR4 (s)	Average TLR4	CD8 (d)	CD8 (s)	Average CD8	p16
PDL‐1 (s)	Correlation coefficient	0.030	0.084	0.060	−0.152	−0.015	−0.134	0.046	0.347**	1	0.661**	0.106	−0.348*	−0.177	−0.215	0.377**	0.384**	0.383**	−0.070
	*p*	0.781	0.447	0.743	0.159	0.893	0.213	0.675	**0.002**		**< 0.001**	0.353	**0.014**	0.200	0.083	**0.001**	**< 0.001**	**< 0.001**	0604
PDL‐1 (d)	Correlation coefficient	0.204	−0.053	−0.020	0.009	−0.159	−0.067	0.142	**1**	0.347**	0.913**	0.065	−0.180	−0.049	−0.098	0.303**	0.246*	0.287**	0.033
	*p*	0.051	0.621	0.913	0.934	0.131	0.523	0.185		**0.002**	**< 0.001**	0.569	0.206	0.736	0.441	**0.005**	**0.026**	**0.007**	0.801
Average PDL1	Correlation coefficient	0.128	0.039	0.112	0.039	0.025	0.056	0.167	0.913**	0.661**	1	0.080	−0.269*	−0.119	−0.139	0.312**	0.235*	0.276**	0.009
	*p*	0.197	0.704	0.486	0.698	0.798	0.573	0.096	**< 0.001**	**< 0.001**		0.456	**0.049**	0.379	0.243	**0.003**	**0.023**	**0.006**	0.943
Ki67%	Correlation coefficient	0.366**	0.046	0.132	0.075	0.056	0.005	0.087	0.065	0.106	0.080	1	−0.168	−0.099	−0.165	−0.028	−0.052	−0.042	0.221
	*p*	**0.000**	0.657	0.437	0.458	0.585	0.958	0.402	0.569	0.353	0.456		0.234	0.465	0.167	0.800	0.628	0.689	0.072
TLR4 (d)	Correlation coefficient	−0.086	−0.147	−0.095	0.129	0.148	0.185	−0.058	−0.180	−0.348*	−0.269*	−0.168	1	0.478**	0.922**	−0.082	−0.112	−0.132	0.073
	*p*	0.502	0.254	0.636	0.312	0.246	0.146	0.655	0.206	**0.014**	**0.049**	0.234		**0.001**	**< 0.001**	0.558	0.416	0.322	0.648
TLR4 (s)	Correlation coefficient	−0.157	−0.173	0.070	0.141	0.123	0.065	0.162	−0.049	−0.177	−0.119	−0.099	0.478**	1	0.791**	−0.248	−0.007	−0.137	0.015
	*p*	0.211	0.176	0.739	0.262	0.329	0.608	0.210	0.736	0.200	0.379	0.465	**0.001**		**< 0.001**	0.071	0.958	0.293	0.921
Average TLR4	Correlation coefficient	−0.107	0.217*	−0.181	0.009	0.001	0.007	0.016	−0.098	−0.215	−0.139	−0.165	0.922**	0.791**	1	−0.135	−0.058	−0.122	0.040
	*p*	0.335	**0.050**	0.314	0.938	0.991	0.953	0.884	0.441	0.083	0.243	0.167	**< 0.001**	**< 0.001**		0.267	0.621	0.289	0.771
CD8 (d)	Correlation coefficient	−0.087	−0.063	−0.157	0.063	0.092	0.004	0.082	0.303**	0.377**	0.312**	−0.028	−0.082	−0.248	−0.135	1	0.640**	0.877**	0.043
	*p*	0.382	0.534	0.326	0.527	0.357	0.969	0.418	**0.005**	**0.001**	**0.003**	0.800	0.558	0.071	0.267		**< 0.001**	**< 0.001**	0.735
CD8 (s)	Correlation coefficient	−0.038	−0.072	−0.340*	0.013	0.118	0.018	0.051	0.246*	0.384**	0.235*	−0.052	−0.112	−0.007	−0.058	0.640**	1	0.932**	0.147
	*p*	0.699	0.468	**0.024**	0.896	0.227	0.853	0.606	**0.026**	**< 0.001**	0.023	0.628	0.416	0.958	0.621	**< 0.001**		**< 0.001**	0.231
Average CD8	Correlation coefficient	−0.058	−0.060	−0.221	0.081	0.108	0,040	−0,002	0.287**	0.383**	0.276**	−0.042	−0.132	−0.137	−0.122	0.877**	0.932**	1	0.118
	*p*	0.538	0.529	0.131	0.391	0.252	0.673	0.979	**0.007**	**< 0.001**	0.006	0.689	0.322	0.293	0.289	**< 0.001**	**< 0.001**		0.321
p16	Correlation coefficient	0.179	−0.033	0.164	0.079	−0016	0.015	0.023	0.033	−0.070	0.009	0.221	−0.073	−0.015	−0.040	−0.043	−0.147	−0.118	1
	*p*	0.081	0.757	0.306	0.447	0.877	0.882	0.836	0.801	0.604	0.943	0.072	0.648	0.921	0.771	0.735	0.231	0.321	

*Note*: Bold values are statistically significant.

Abbreviations: Average = mean between (s) and (d); (d) = deep invasion front of the tumor cells; Dimension = maximum size of primary tumor (< 2 cm vs. > 2 cm); DOI = deep of invasion (< 10 mm vs. > 10 mm); G = Grade (G1 VS G2/G3); (s) = superficial tumor samples; Stage = I‐II vs. III‐IV; T = T1‐T2 early‐stage vs. T3‐T4 advanced stage; *p < 0.05; **p < 0.001.

### 3.3 Clinical Outcomes

A logistic regression was performed to assess the impact of each variable on OSCC mortality (Table 4). The univariable analysis revealed that patients with advanced T stage (T3‐T4 > 10 mm) have 6.6 higher chances of mortality due to their tumor than early T staged (T1‐T2 5–10 mm) (HR: 6.61, 95% CI 2.8–15.6, *p* = 0.0001). Similarly, higher tumor grades increased the likelihood of death. In addition, lymph nodes' metastatic involvement (N+) results in a 5.85 higher risk of mortality than N0 (HR: 5.85, 95% CI 2.5–13.5, *p* = 0.0001). Furthermore, for OSCC cases that were treated, besides surgery, with chemotherapy alone or in combination with radiotherapy, there were 44 more chances of death (HR: 44.96, 95% CI 3.7–32.2, *p* = 0.0001). Multivariate Cox proportional hazard model analysis was performed assessing the significant variables in the univariate analysis as the multivariate model revealed that advanced T‐staged tumors (HR: 5,16, 95% CI 1,7‐14,8, *p* = 0,002), lymph node metastasis (HR: 4.0, 95% CI 1,4‐11,03, *p* = 0,007) and receiving chemotherapy (HR: 9.7, 95% CI 2,9‐32,2, *p* = 0,0001) were significant independent risk factors for OSCC mortality.

**TABLE 4:** Univariate and multivariate analyses of overall survival of patients based on clinicopathological features and the expression of Ki‐67, TRL4, PD‐L1 and CD8.

		Univariate			Multivariate		
		HR	95% CI	*p*	HR	95% CI	*p*
Gender	Female	Ref					
	Male	0.94	0.407–2.209	0.903			
Tumor grade	Grade 1	Ref					
	Grade 2	3.46	1.017–11.817	**0.047**	3.31	0.602–18.209	0.17
	Grade 3	4.72	1.215–18.388	**0.025**	2.18	0.339–14.072	0.41
T	T1^a^ + T2^a^	Ref					
	T3^a^ + T4^a^	6.61	2.800–15.608	**0.0001**	5.16	1.796–14.813	**0.002**
N	N0	Ref					
	N +	5.85	2.536–13.534	**0.0001**	4.00	1.452–11.033	**0.007**
Adjuvant treatment	Chm	44.968	3.774–32.204	**0.0001**	9.73	2.937–32.247	**0.0001**
	No Chm	Ref					
	RTx	1.88	0.714–4.960	0.201			
	No RTx	Ref					
p16	p16 −	0.52	0.216–1.295	0.163			
	p16 +	Ref					
Ki‐67	% positive	0.98	0.973–1.005	0.171			
TLR4%	*(d)*	1.013.00	0.994–1.032	0.196			
	*(s)*	1.006.00	0.986–1.026	0.567			
PD‐L1%	Average	0.988	0.967–1.009	0.246			
	*(d)*	0.990	0.965–1.015	0.438			
	*(s)*	0.967	0.935–1.00	0.053			
CD8	Cell + / mm^2^ *(d)*	1.000	1.000–1.000	0.678			
	Cell + / mm^2^ *(s)*	1.000	0.999–1.000	0.181			
	Average	0.999	0.997–1.001	0.267			

*Note*: Bold values are statistically significant.

Abbreviations: CI = confidence interval; HR = hazard ratio.

^a^ According to AJCC 8th edition 2017 edition.

## 4 Discussion

The role of inflammation in carcinogenesis has been extensively studied. Investigating Toll‐like receptors and the PD‐L1 pathway has advanced understanding of tumorigenesis. Characterizing the tumor microenvironment's immune response is vital for understanding immune cell roles in cancer initiation and progression, particularly in oral cancer. PD‐L1 expression on oral tumor cells may adaptively respond to TLR4‐mediated inflammation, causing CD8+ TIL exhaustion and immune evasion. Interestingly, our study detected an inverse correlation among these immune markers in a large OSCC cohort.

TLR4 is a receptor expressed on innate immune cells such as macrophages, neutrophils, and lymphocytes. It recognizes pathogen‐associated molecular patterns (PAMPs) and damage‐associated molecular patterns (DAMPs) [11]. Binding of PAMPs or DAMPs to TLR4 triggers an intracellular signaling cascade involving adapter molecules like MyD88 and TRIF, activating transcription factors such as NF‐κB and IRF3. This results in the production of pro‐inflammatory cytokines (e.g., TNF‐α, IL‐6, IL‐1β) and type I interferons [18, 19]. TLR's role in cancer balances pro‐inflammatory antitumor effects and promotion of a chronic inflammatory, pro‐tumor environment [18]. Our findings show no correlation between TLR4 and cell cycle progression or invasive capability, consistent with Li et al., who found that reducing TLR4 expression disrupts cell survival and decreases inflammatory cytokine production [19].

PD‐L1 is a protein crucial for regulating immune responses, particularly in cancer. By suppressing the adaptive immune response, it enables tumor cells to evade apoptosis by effector cells [7]. Expressed on tumor and immune cells, PD‐L1 binds to the PD‐1 receptor on activated T cells, B cells, and NK cells; this interaction inhibits T cell activation and proliferation, reduces effector cytokine production (e.g., IFN‐γ), and decreases T cell cytotoxic activity. Once activated, this immune checkpoint suppresses the antitumor immune response [20].

Although TLR4 and PD‐L1 are key regulators of inflammation and immune response, their interplay remains unclear. In colorectal cancer, PD‐L1 expression was significantly associated with TLR4, with high levels of both correlating with poor disease‐free survival [21]. Similarly, Kang et al. (2020) reported a positive correlation between TLR4 and PD‐L1 expression, with higher levels linked to worse prognosis in non‐small cell lung cancer [16]. However, our study found an inverse correlation between PD‐L1 and TLR4 in OSCC, possibly due to their contrasting roles in tumor immunology. TLR4 activation typically promotes pro‐inflammatory signaling, potentially enhancing anti‐tumor immunity through the activation of immune cells such as macrophages and dendritic cells. In contrast, PD‐L1 expression is a key mechanism by which tumors evade immune surveillance, suppressing T‐cell activity via the PD‐1/PD‐L1 axis. An inverse relationship might arise due to shifts in the tumor's immune environment. Elevated TLR4 expression could sustain pro‐inflammatory responses that counteract the immune‐suppressive pathways mediated by PD‐L1. Conversely, tumors with high PD‐L1 levels may suppress the inflammatory pathways associated with TLR4 to establish an immune‐tolerant environment conducive to tumor growth [12].

The positive correlation between PD‐L1 and CD8 found in this study has also been observed in other studies related to other types of cancer, such as non‐small cell lung cancer [22], intrahepatic cholangiocarcinoma [23], intestinal gastric adenocarcinoma, invasive breast carcinoma of no special type, and liposarcoma [24]. Different mechanisms can explain the positive association between the cytotoxic CD8 T lymphocytes and PD‐L1 expression on tumor cells. Cytotoxic T cells may recognize tumor cells and produce interferon*‐γ*; PD‐L1 expression is in turn induced by interferon‐γ, leading to evasion of the T cell response [22].

The abundance of CD8+ T lymphocytes in the tumor microenvironment is linked to better outcomes in cancers such as gastric, non‐small cell lung, and esophageal cancers [25, 26]. These lymphocytes eradicate tumor cells through cytotoxic activity and regulate antitumor immunity, often correlating with reduced tumor progression, lower recurrence rates, and improved survival. A meta‐analysis showed that high CD8 expression in esophageal cancer is associated with longer overall and disease‐free survival [26]. However, in our OSCC cohort, CD8 positivity was not linked to better survival. Instead, higher CD8+ TIL density was observed in advanced tumors (T3, T4). This suggests increased CD8+ infiltration does not always indicate improved immune response and that additional factors may be involved. The positive correlation with PD‐L1 levels may indicate functional impairment, as interactions with PD‐L1 potentially deactivate CD8+ cells. T‐cell exhaustion, driven by chronic inflammation and characterized by inhibitory receptor upregulation, reduced cytokine production, and impaired cytotoxicity, likely underlies this effect [27]. PD‐1, the primary inhibitory receptor, mediates this exhaustion, rendering T cells ineffective against cancer cells [27]. Thus, assessing CD8+ TILs as a prognostic biomarker requires considering PD‐L1 levels.

Expression of PD‐L1 was more intense in more advanced grades of the disease (grade 3, *p* = 0.031). The study by Gulinac et al. (2020) also brings similar results in which PD‐L1 expression in higher grades of OSCC was 61% compared to 14% in lower stages, just as PD‐L1 expression in OSCC was higher than in potentially malignant disorders, showing that a greater expression of PD‐L1 is acquired with tumor progression. In bladder cancer, positive expression of PD‐L1 was also associated with high grade and high pathological status [28]. As in a study on ovarian cancer in which Grade 3 cancers presented higher PD‐L1 positivity [29].

In the analysis of Ki‐67 concerning cancer grade, a highly significant result was attained; tumors classified as grade 3 histologically presented with significantly higher Ki‐67 index. This discovery implies a consequential correlation between Ki‐67 levels and cancer progression, underscoring the potential applicability of this marker in assessing and characterizing distinct stages of the disease. In this study, a significant increase in the risk of death is demonstrated in patients treated with chemotherapy. This, on the one hand, confirms the consistent ineffectiveness of traditional chemotherapy treatments in advanced stages of OSCC progression, but on the other hand, this effect is partly due to potential bias due to a retrospective study in which patients treated with chemotherapy had a greater risk of mortality (Advanced T Stage, positive surgical margins, *N*+ positive status) at the time of treatment.

In conclusion, this study demonstrated an inverse correlation between the expression of PD‐L1 and TLR4 at deep and superficial tumor fronts, suggesting that it may identify distinct immunological groups. Furthermore, PD‐L1 expression was associated with a greater number of CD8+ cells; however, these factors were not significant in determining mortality risk. These results suggest that there is a differential regulation of the immune response coordinated by activation of PD‐L1 or TLR4. Accurate characterization of these markers can guide the development of more effective immunotherapies. Immune checkpoint inhibitors, such as anti‐PD‐1 and anti‐PD‐L1, have shown promising potential to reverse CD8+ T cell exhaustion and restore immune surveillance against oral tumors. Furthermore, TLR4 modulators can be explored to balance the inflammatory response and reduce immunosuppression within the tumor microenvironment. Specifically, PD‐L1‐negative cases may be candidates for therapies aimed at countering cell survival mechanisms such as immortalization and apoptosis inhibition. These mechanisms, driven by innate immune activation, enable cancer cells to survive in hostile environments, such as those created by chronic inflammation or infection.

## Ethics Statement

This study was performed in accordance with good clinical practice guidelines and the Declaration of Helsinki and with the prior approval of the ethics committee of the University of Palermo and the Azienda Ospedaliera Universitaria Policlinico Giaccone–Palermo (Ethics Committee: Prot. N. 11/2011).

## Conflicts of Interest

The authors declare no conflicts of interest.

## Peer Review

The peer review history for this article is available at https://www.webofscience.com/api/gateway/wos/peer‐review/10.1111/jop.70012.

## Supporting information


**Figure S1:** Expression levels of TLR4 in 166 OSCC cases, grouped according to clinicopathological features. (A–D) Quantification of TLR4‐positive cells in superficial tumor areas. (E–H) Quantification of TLR4‐positive cells in deeper tumor areas.

**Figure S2:** Expression levels of PD‐L1 in 166 OSCC cases, grouped according to clinicopathological features. (A–D) Quantification of PD‐L1‐positive cells in superficial tumor areas. (E–H) Quantification of PD‐L1‐positive cells in deeper tumor areas.

**Figure S3:** Quantification of CD8^+^ tumor‐infiltrating lymphocytes in 166 OSCC cases, grouped according to clinicopathological features. (A–D) Quantification of CD8^+^ TILs in superficial tumor areas. (E–H) Quantification of CD8^+^ TILs in deeper tumor areas.

## Data Availability

The data that support the findings of this study are available from the corresponding author upon reasonable request.
