# Supplementary figures and images for: Inverse Regulation of TLR4 and PD‐L1 Shapes the Inflammatory Tumor Microenvironment in Oral Squamous Cell Carcinomas
Source: J Oral Pathol Med. 2025 Aug 5;54(8):676–86. doi: 10.1111/jop.70012 (PMC12419984; doi:10.1111/jop.70012)

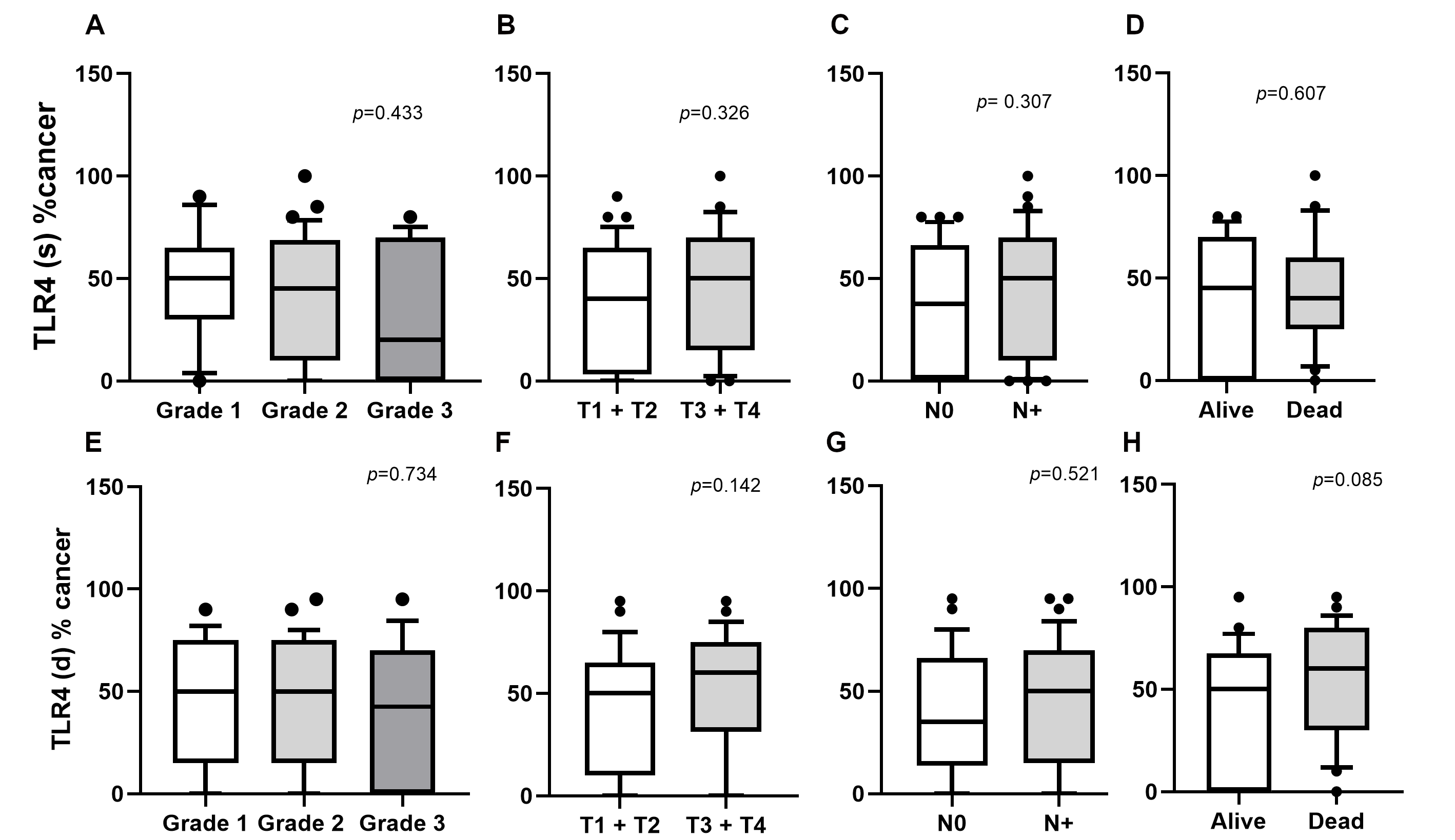

Supplement: Supplementary file 1 — Figure S1: Expression levels of TLR4 in 166 OSCC cases, grouped according to clinicopathological features. (A–D) Quantification of TLR4‐positive cells in superficial tumor areas. (E–H) Quantification of TLR4‐positive cells in deeper tumor areas. [file JOP-54-676-s002.tif]

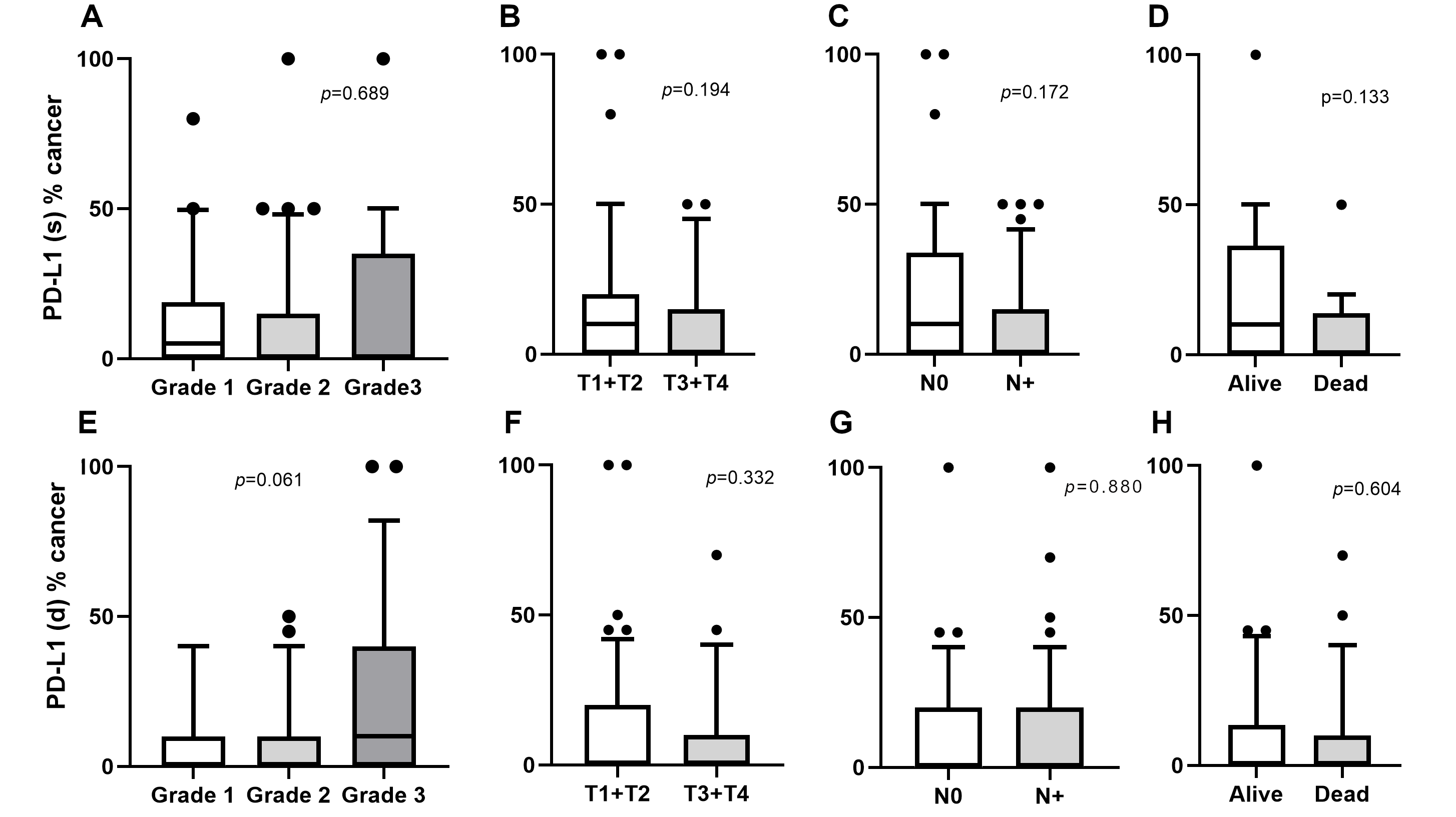

Supplement: Supplementary file 2 — Figure S2: Expression levels of PD‐L1 in 166 OSCC cases, grouped according to clinicopathological features. (A–D) Quantification of PD‐L1‐positive cells in superficial tumor areas. (E–H) Quantification of PD‐L1‐positive cells in deeper tumor areas. [file JOP-54-676-s001.tif]

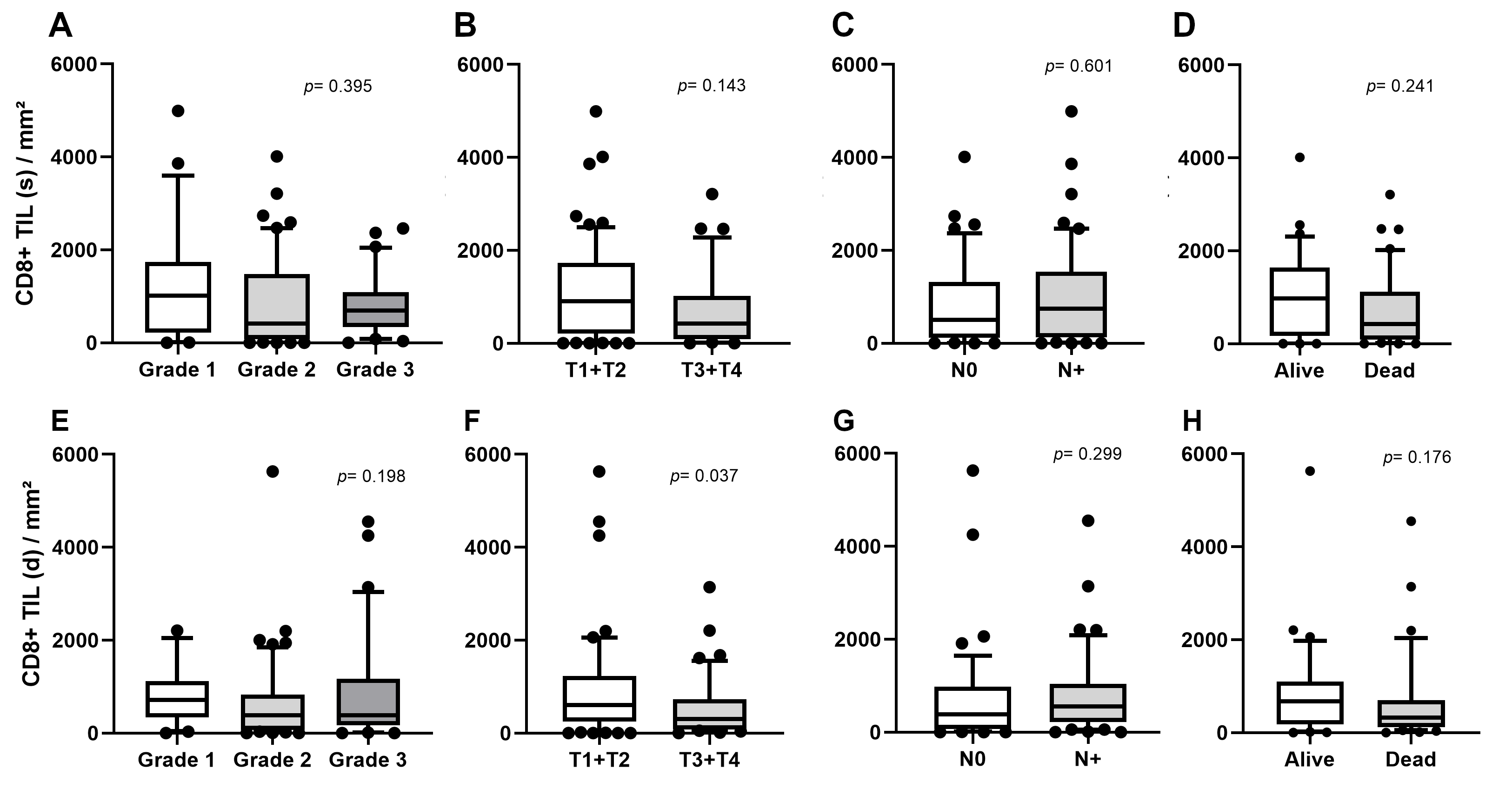

Supplement: Supplementary file 3 — Figure S3: Quantification of CD8+ tumor‐infiltrating lymphocytes in 166 OSCC cases, grouped according to clinicopathological features. (A–D) Quantification of CD8+ TILs in superficial tumor areas. (E–H) Quantification of CD8+ TILs in deeper tumor areas. [file JOP-54-676-s003.tif]
